# A new chronobiological approach to discriminate between acute and chronic depression using peripheral temperature, rest-activity, and light exposure parameters

**DOI:** 10.1186/1471-244X-13-77

**Published:** 2013-03-09

**Authors:** Cláudia Ávila Moraes, Trinitat Cambras, Antoni Diez-Noguera, Regina Schimitt, Giovana Dantas, Rosa Levandovski, Maria Paz Hidalgo

**Affiliations:** 1 Laboratório de Cronobiologia do Hospital de Clínicas de Porto Alegre (HCPA), da Universidade Federal do Rio Grande do Sul (UFRGS), Ramiro Barcelos, 2350 sala 12107, Porto Alegre, RS, 90035-003, Brazil; 2 Departament de Fisiologia, Facultat de Farmàcia, Universitat de Barcelona, Avinguda de Joan XXIIIs/n, Barcelona, 08028, Spain; 3 Programa de Pós-Graduação em Ciências Médicas: Psiquiatria, UFRGS, Porto Alegre, Brazil; 4 Departamento de Psiquiatria e Medicina Legal da Faculdade de Medicina, da Universidade Federal do Rio Grande do Sul, Porto Alegre, Brazil

**Keywords:** Depression, Temperature, Activity, Light, Circadian rhythm

## Abstract

**Background:**

Circadian theories for major depressive disorder have suggested that the rhythm of the circadian pacemaker is misaligned. Stable phase relationships between internal rhythms, such as temperature and rest/activity, and the external day-night cycle, are considered to be crucial for adapting to life in the external environmental. Therefore, the relationship and possible alterations among (i) light exposure, (ii) activity rhythm, and (iii) temperature rhythm could be important factors in clinical depression. This study aimed to investigate the rhythmic alterations in depression and evaluate the ability of chronobiological parameters to discriminate between healthy subjects and depressed patients.

**Methods:**

Thirty female subjects, including healthy subjects, depressed patients in the first episode, and major recurrent depression patients. Symptoms were assessed using Hamilton Depression Scale, Beck Depression Inventory and Montgomery-Äsberg Scale. Motor activity, temperature, and light values were determined for 7 days by actigraph, and circadian rhythms were calculated.

**Results:**

Depressed groups showed a lower amplitude in the circadian rhythm of activity and light exposure, but a higher amplitude in the rhythm of peripheral temperature. The correlation between temperature and activity values was different in the day and night among the control and depressed groups. For the same level of activity, depressed patients had lowest temperature values during the day. The amplitudes of temperature and activity were the highest discriminant parameters.

**Conclusions:**

These results indicate that the study of rhythms is useful for diagnosis and therapy for depressive mood disorders.

## Background

Depression is among the most prevalent, debilitating, and potentially fatal mental disorders [1,2]. The onset of symptoms is sex-dependent, with the highest prevalence in females. Experimental evidence suggests that during development, alterations in the medial amygdala cause sexual dimorphism in the expression level of an evolutionarily conserved pathway that affects mitochondrial and circadian clock function [3]. Mitochondrial and circadian clock function are associated with mood disorders [4].

The brain's primary circadian pacemaker is the suprachiasmatic nucleus (SCN), which is synchronized by light input from melanopsin-containing retinal ganglion cells, and consecutively its system synchronizes subsidiary oscillators [4,5]. Changes in SCN-dependent rhythms have been observed in patients with mood disorders [6]. Therefore, depressive symptoms can be a consequence of an altered response to input of environmental cues, such as light exposure [7].

Depressive symptoms improve after light therapy [8]. The efficacy of bright light therapy as an adjuvant treatment to antidepressant pharmacotherapy in nonseasonal depression has also been demonstrated [9]. Studies suggest that bright light therapy alone is more efficacious for seasonal affective disorder than it is for nonseasonal depression [9], probably because there are different levels of SCN involvement in the aetiology of depressive mood.

In depressive patients, changes in the time of light exposure and social life show greater variability and are prone to desynchronization in rest-activity and temperature [10]. One of the first circadian theories for major depressive disorder suggested that the circadian pacemaker is misaligned with respect to the timing of sleep, as well as to the activity-rest rhythm [11,12]. This evidence combined with the clinical observation that altered physical activity has long been recognized as a feature of depression [13], indicate that this variable could be one of the main parameters involved in mood disorders [14].

Other characteristics of patients with depressive episodes include the presence of daytime mood variations, and alterations in the circadian secretion of cortisol and adrenocorticotropic hormone [6]. Exogenous administration of human corticotropin-releasing hormone provokes significant phase-advancements in body temperature [15], one of the most robust circadian rhythms [16]. Abnormal body temperature variation, which has been observed in depressed patients, could be considered as a functional disturbance located at the level of circadian organization [17].

Stable phase relationships between internal rhythms, such as temperature and rest/activity, and the external day-night cycle, are considered important for adapting to life in the external environmental [18,19]. Therefore, the relationship and possible alterations among (i) light exposure, (ii) activity rhythm, and (iii) temperature rhythm could be important factors in clinical depression. We hypothesized that these three variables are disrupted in depression and that they might be important for the evaluation of clinical depression. Therefore, we designed a cross-sectional study to examine this chronodisruption in depression, and to evaluate the capacity of chronobiotic parameters to discriminate between healthy subjects, depressed patients in their first episode without previous treatment, and patients with major recurrent depression.

## Methods

### Sample characteristics

The investigation followed a cross-sectional design and was conducted in the city of Porto Alegre, which is located in the extreme south of Brazil (30° 05' South and 51° 10' West). This study was performed according to international ethical standards [20] and was conducted in accordance with the Declaration of Helsinki. The study protocol (number 06-268 GPPG/HCPA) was approved by the Ethics Committee at the Hospital de Clínicas de Porto Alegre (HCPA), and all participants signed an informed consent form.

**Figure 1:**
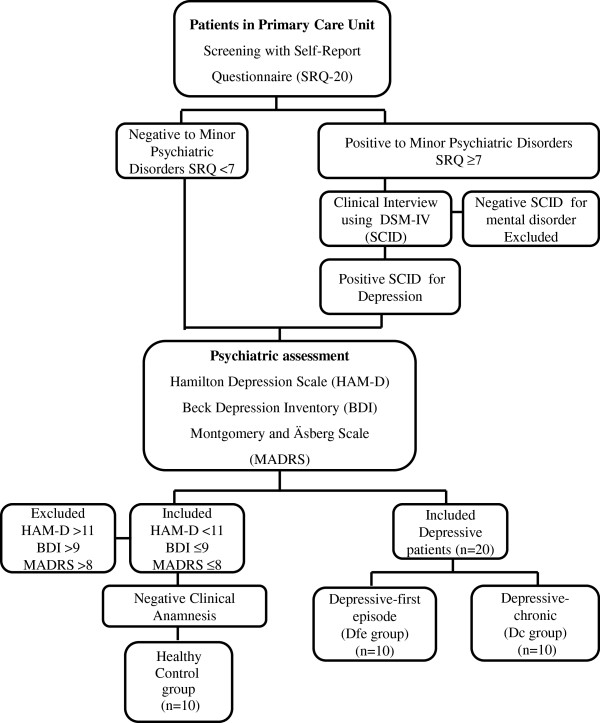
Flowchart of the study protocol.

The flowchart of the study protocol is shown in Figure 1. Thirty female patients aged between 18 and 60 years (10 controls and 20 depressed patients) were selected using the Self-Report Questionnaire (SRQ-20), which was used to assess minor psychiatric disorders at the municipal primary health care units. The volunteers were referred for an additional assessment by psychiatrists in the psychiatric outpatient clinic at the hospital (HCPA). The Structured Clinical Interview for DSM-IV (SCID) was conducted to diagnose depression.

Ten patients met the criteria for a first depressive episode; they had not used antidepressants previously (depressive-first episode: D_fe_ group). Ten patients were diagnosed with major recurrent depression (mean number of episodes = 3) and were using antidepressants (depressive-chronic: D_c_ group).

The subjects in the control group (10 healthy subjects) were matched by age with those in the depressive groups. All the exclusion criteria for the depressive groups were applied to the control group. Moreover, exclusion criteria for the control group also included the presence of symptoms meeting criteria for diagnosis of a mood disorder, the presence of greater than mild symptoms on any depression scale, and the use of psychopharmacological drugs.

None of the patients had performed shift work or had transmeridian travel during the month preceding the study. Exclusion criteria were the use of beta-blockers or diuretics, the presence of severe psychiatric co-morbidity, and the following standard exclusion criteria for clinical trials: pregnancy, chronic clinical disorder, and alcohol or illicit drug abuse.

To avoid bias in measurements, trained interviewers assessed depressive symptoms using the following questionnaires validated in Brazil: the Hamilton Depression Scale (HAM-D) [21,22], the Beck Depression Inventory (BDI) [23,24], and the Montgomery-Äsberg Scale (MADRS) [25]. The participants were blinded to the objective of the study.

### Instruments

#### Assessment of minor psychiatric disorders

Minor psychiatric disorders were assessed with the SRQ-20, adapted by Mari and Williams [2]. The presence of minor psychiatric disorders was defined by an SRQ-20 score ≥7 for women. Using these cut-off values, the sensitivity of the method to detect minor psychiatric disorders is 83.5%, and the specificity is 80% [2].

### Assessment of depression

The HAM-D scale includes 21 questions, with the final score ranging from 0 to 62 [21]. Patients with HAM-D scores of 11 to17 were classified as having mild depression, 18 to 25 as moderate depression, and >25 as having a severe depressive episode [22].

The BDI is a self-report scale that can be used as a screening questionnaire to assess the cognitive, affective [23], and somatic symptoms of depression [24]. The BDI includes 21 items; each response is assigned a score ranging from 0 (no symptoms of depression) to 3 (severe symptoms of depression). The total score ranges from 0 to 63. Total scores from 0 to 9 points indicate no or minimal depressive symptoms, 10 to 16 points indicate mild depressive symptoms, 17 to 29 points indicate moderate depressive symptoms, and 30 to 63 points indicate severe depressive symptoms, as described previously [24].

The MADRS scale contains 10 questions that are each scored from 0 to 6 points. The final score ranges from 0 to 60 points [25] An intra-class correlation coefficient of 0.96 was observed for the agreement between the different evaluators. Individuals with scores <7 were classified as being without depressive symptoms.

### Assessment of temperature, activity, and light rhythms

The rhythm variables were assessed by continuous actigraphy monitoring with light sensors in all patients over 7 days. The actigraph model used in this study was a Tempatilume® (Cebrasil, Inc. Brazil), which measures activity, peripheral temperature, and ambient light exposure. The Tempatilume is an activity monitor designed for the long term monitoring of activity in human subjects. This monitor contains an accelerometer that is capable of sensing any motion with a minimal resultant force of 0.01 g. All communication with the actigraph is accomplished using a Tempatilume reader® (Cebrasil, Inc.) that is connected to a computer via a USB port. The epoch length was 1 minute. The software used was Tempatilume Rhythm Analysis® (Cebrasil, Inc.). The data were converted into ASCII files that could be processed by the chronobiology program “El Temps”. The subjects wore wrist actigraphs with light sensors for 7 days.

### Data processing and analysis

Motor activity and peripheral temperature data were first visually inspected by double-plotted graphs. The circadian rhythm was examined by the periodogram of Sokolove and Bushell [26], which provides the period of the most significant rhythm and the percentage of variance (PV) explained by this rhythm. PV was used as a marker of the stability and prominence of the rhythm. Periods and PV were considered significant at a level of p < 0.05. Moreover, for each patient, data were fitted to a cosinusoidal curve of a 24-hour rhythm (cosinor analysis). This analysis provided the amplitude, mesor, and acrophase of the adjusted rhythm. The mean and standard errors of the daily acrophases were calculated. The stability of the daily phases of each individual circadian rhythm was calculated by the Rayleigh z test. The resulting vector angle indicated the mean peak time for each individual rhythm, and the magnitude of the vector (r) indicated the phase coherence. Therefore, the higher the r, the more coherent the phase of the rhythm was for this individual. To study the phase tendencies of each group, Rayleigh z tests were used in relation to the individual vectors, as described above, to obtain the mean vector of each group.

The mean daily profiles of the rhythm of each variable and for each patient were calculated. From these profiles, we calculated the mean daily values of motor activity, peripheral temperature, and light, as well as the diurnal and nocturnal mean values of each of the three variables. We also calculated the time that the variable was above the mean.

In addition, to study the relationship between temperature and activity rhythms in each subject, the activity data were transformed into percentiles for each individual (10 levels were calculated based on the maximum activity of each individual), and the temperature values corresponding to each activity level were determined. The relationships between activity and temperature were plotted separately for day and night. This methodology has been described elsewhere [27,28].

### Statistical analysis

Data are expressed as the mean ± SEM. The calculated variables were tested using one-way analysis of variance (ANOVA). We also estimated statistic partial Eta squared to measure the effect size 0.04 a medium effect and 0.1 a large effect size, where 0.01 constitutes a small effect, 0.04 a medium effect and 0.1 a large effect. All post-hoc comparisons were Bonferroni corrected. The rhythm variables derived from the cosinor analysis, amplitude (difference between the mesor and peak), and acrophase (clock time of the peak value), were also evaluated by discriminant analysis to estimate which variable (light, activity, or temperature rhythm) presented higher coefficients to discriminate patients in the D_c_ group from those in the D_fe_ group, and the control group from both depressive groups. For all analyses, the statistical significance was set at p < 0.05, with a two-tailed hypothesis. When only two means were compared, data were tested by independent Student’s t-tests. Data were analysed using SPSS version 16.0 (SPSS, Chicago, IL, USA).

## Results

Demographic characteristics among the depressive patient groups are shown in Table 1. Although the patients were taking medication, the D_c_ group had higher scores than those in the D_fe_ group in the BDI and HAM-D, but not with the MADRS instrument. Also, there was no significant age difference among groups (F (df) = 0.17(2); P > 0.05).

**Table 1:** Differences in demographic characteristics between the depressive disorder groups

	**D**_**fe**_	**D**_**c**_	**Student *t *test for independent samples**	
	**Mean ± SE**	**Mean ± SE**	***T***	***P***
Age	44 + 3.94	44.82 + 3.33	0.16	0.88
Beck	17.90 + 2.8	32.27 + 4.3	-2.78	0.013
Hamilton	17.40 + 4.8	25.18 + 9.27	-2.45	0.027
Montgomery-Asberg	21.5 + 5.08	23.00 + 9.76	-0.5	0.66

*D*_*fe*_ Depressives-first episode, *D*_*c*_ Depressives-chronic, *SE* Standard error of the mean.

**Figure 2:**
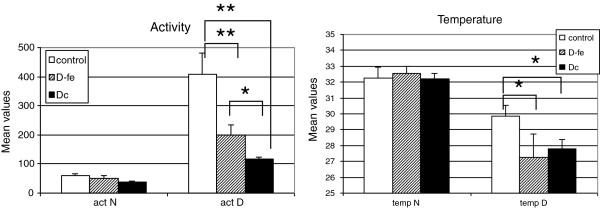
**Mean levels of motor activity and peripheral temperature during the day (D) and night (N) for each group (D**_**fe**_**: depressive-first episode, D**_**c**_**: depressive-chronic).** **p < 0.001; *p < 0.05.

When the mean values for motor activity and temperature for day and night were calculated for the three groups (Figure 2), motor activity had the highest values during the day, and peripheral temperature had the highest values during the night, as expected. For motor activity and temperature, differences between the groups were found in diurnal values, but not in nocturnal values. Diurnal activity was significantly higher in the control group than in the depressive groups (ANOVA, F (df) = 23.63(2); p < 0.001; eta-squared = 0.673). Post-hoc analysis showed that differences were greater between control and D_fe_ (p < 0.0001) than under control and D_c_ (p < 0.01), and also that diurnal activiy was higher in D_fe_ than in D_c_ (p < 0.05). With regard to peripheral temperature, although diurnal values appeared significantly higher in the control group than in the depressive groups, ANOVA did not show statistically significant differences, probably due to the high dispersion in the values of D_fe_ group; nevertheless, Student’s t test show differences between control and D_c_ (Student’s t test, t = 2.346, p < 0.05).

**Figure 3:**
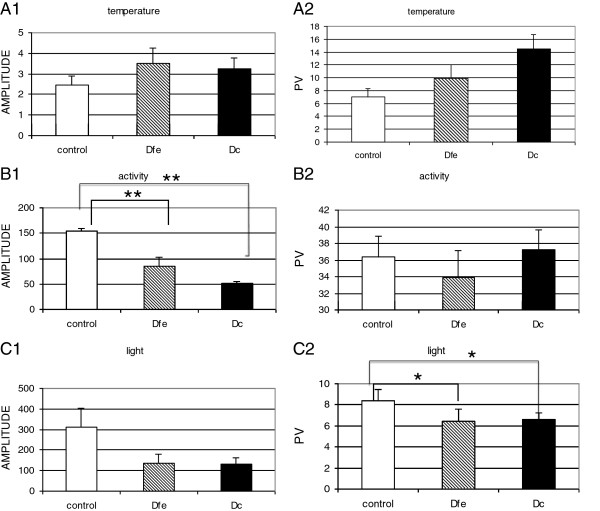
**Amplitude values (left) and PV explained by the 24 h rhythm obtained by the Sokolove and Bushell periodogram (right).** Standard errors for the activity, peripheral temperature, and light exposure of each group (D_fe_: depressive-first episode; D_c_: depressive-chronic; PV: percentage of the variance) are shown.

Study of the amplitude of the rhythm, obtained by cosinor analysis (Figure 3A1, B1, C1), showed that the amplitude values for activity were significantly higher in the control group than in either of the depressive groups (ANOVA, F(df) = 23.72(2); p < 0.001; eta-squared = 0.673). Post-hoc tests also indicate that amplitude values for activity in the D_fe_ group compared with those in the D_c_ group were close to significance (p = 0.08). The amplitude of the temperature rhythm was not different between the groups. The amplitude of the light intensity was higher in control than in depressive groups (ANOVA, F(df) = 3.729(2); p < 0.05; eta-squared = 0.245). No differences were found in light intensity in the depressive groups.

The PV of activity and temperature values explained by the circadian rhythm were not different between the groups (Figure 3A2, B2, C2), although in the control group, temperature tended to be slightly lower than in the depressive groups (ANOVA, F(df) = 2.697(2); p = 0.088; eta-squared = 0.183). The PV of light intensity values was higher in the control group (ANOVA, F(df) = 6.69(2); p < 0.01; eta-squared = 0.358) than in either of the depressive groups, indicating more stability and higher values of this variable in the controls.

**Figure 4:**
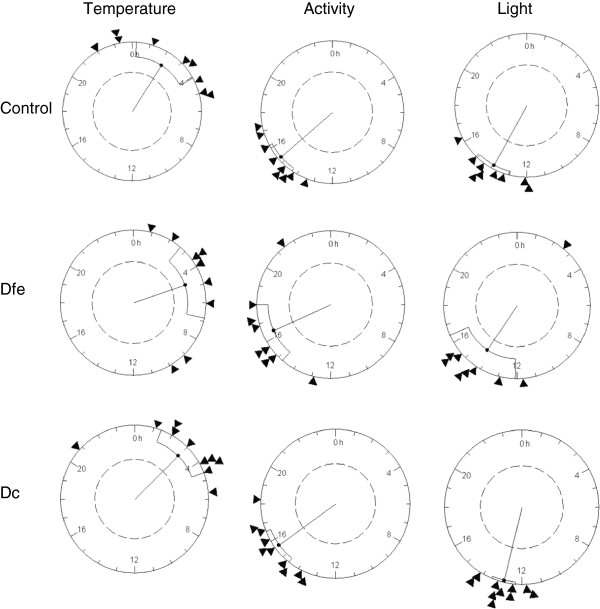
**Rayleigh z tests for activity, peripheral temperature, and light exposure.** Each circle represents 24 hours, and each triangle indicates the position of the mean acrophase of each individual in the group. The black point of the inside vector represents the mean acrophase, and the fiducial limits are also indicated. The size of the vector indicates the grouping of the acrophases. The internal circle indicates a significant level of *p* = 0.05.

To determine if there were differences in acrophase values of the three variables among the three groups, comparison of acrophases (expressed in radians) was conducted by Rayleigh analysis (Figure 4). No differences were observed in acrophase among the groups for temperature, activity, or light. As expected, light exposure and activity rhythms had similar acrophase values, which were opposite to the acrophase value for peripheral temperature.

Analysis of the daily profile indicated that the time when temperature was above the mean was shorter in the control group than in the depressive groups (mean, control: 8 h and 15 min ±26 min; D_fe_: 9 h and 36 min ± 47 min; D_c_: 10 h and 16 min ± 27 min), although significant differences were only found between the control and D_c_ groups (Student’s *t*-test, p < 0.05). No differences were found in the time above the mean for light and activity.

**Figure 5:**
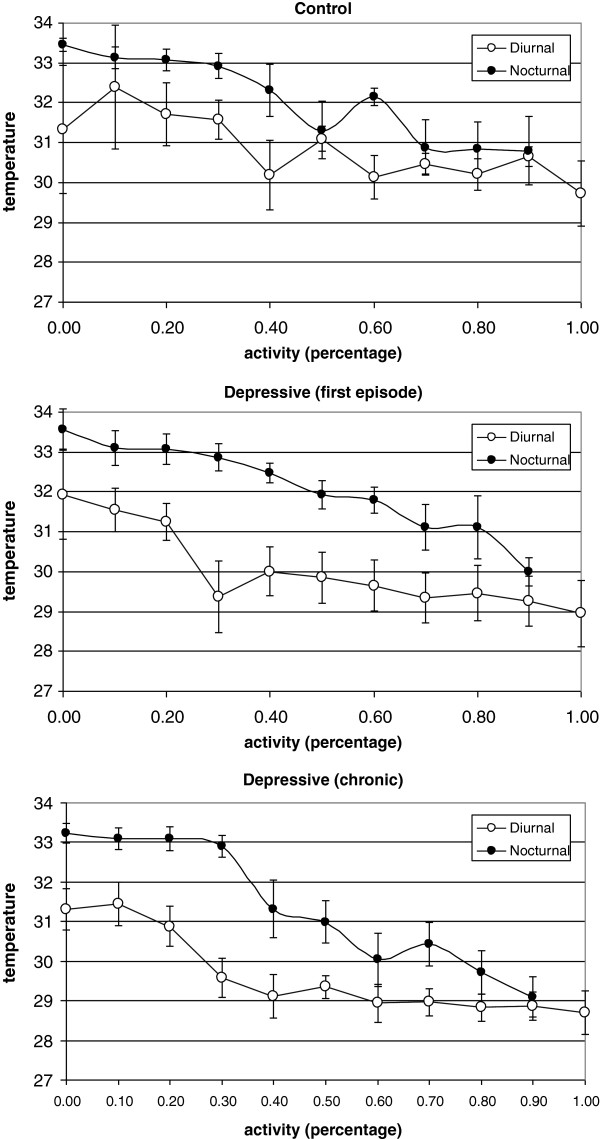
Mean levels of peripheral temperature (diurnal and nocturnal) expressed as a function of activity level (expressed as the percentage of the maximum activity for each group).

The relationships between temperature and the level of activity for each individual differed from day to night. In the day and at night, peripheral temperature decreased as activity increased (Figure 5). However, for the same level of activity, nocturnal temperatures were higher than those obtained during the day, reflecting the circadian rhythm. It is worth noting that the healthy subjects showed less contrast in temperature values between day and night than did the depressive patients. There were no differences between the two groups of depressive patients (Figure 5).

The results of the discriminate analyses are shown in Table 2. The high discriminant coefficients (> 0.3) were branched. The control group could be discriminated from the D_fe_ group by high coefficients of the amplitudes and acrophases of temperature and activity, with the amplitude of temperature as the highest discriminant parameter. The chronic depression group was differentiated from the control and the D_fe_ groups by the activity amplitude.

**Table 2:** **Discriminate analysis of rhythm parameters (amplitude and acrophase) among the control group and the depressive groups (D**_**fe **_**and D**_**c**_**)**

**Variable**	**Control *vs *D**_**fe**_	**Control *vs *D**_**c**_	**D**_**fe **_***vs *D**_**c**_
Amplitude			
Temperature	0.820	-0.269	0.240
Activity	-0.664	0.912	0.398
Light	-0.096	0.178	0.252
Achrophase			
Temperature	-0.442	0.120	-0.111
Activity	0.373	-0.104	0.174
Light	0.039	-0.047	-0.053

*D*_*fe*_ Depressives-first episode, *D*_*c*_ Depressives-chronic, Amplitude: difference between mesor and peak; Acrophase: clock time of the peak value.

## Discussion

In our study, we demonstrated differences in rest-activity, peripheral temperature, and light intensity rhythms in depressive patients compared with healthy subjects. Additionally, we described the capacity of chronobiological variables to discriminate among the different stages (acute and chronic) of a depressive disorder.

With regard to the rest-activity rhythm, we observed that patients in their first depressive episode showed a decrease in amplitude compared with the control group. The amplitude was also reduced in patients with chronic depression. The differences in amplitude might have been due to the subjects’ different activity levels because these differences were reduced according to the severity of the illness [29]. Because this variable was found to be a high coefficient for discriminating chronic depressive patients, it could be an important tool in clinical practice to evaluate the stage and prognosis of major depressive disorder. The increased circadian amplitude in the rest-activity rhythm of healthy subjects could also be explained by the increased entrainment to external Zeitgebers, most likely by the maintenance of robust social and biological Zeitgebers that are essential for life [30].

In the current study, we found that the duration of time that the temperature remained above the mean was higher in the depressive groups than in the control group, suggesting that this variable is a good indicator of the time that subjects might have been in bed or had a low activity level. Activity level is decreased with the severity of the pathology. Several hypotheses have been proposed attempting to relate circadian differences with mood disorders [31], including the phase advance hypothesis, and reduced amplitudes in depressed patients. Findings from previous studies on the amplitude of the temperature rhythm in depressed patients have been inconsistent, perhaps reflecting differences in the samples of depressed patients studied due to differences in the stage or aetiology of the disorder. Therefore, an elevated nocturnal temperature has been reported by some authors [32,33], whereas a blunted or unchanged temperature rhythm has been observed by others [34].

In our study, the amplitude of the activity rhythm was capable of discriminating healthy people from acutely depressed patients. This result might be a marker of the beginning of the disorder because the coefficient of the amplitude of activity presented as the highest discriminant value (0.8). However, the amplitude of peripheral temperature was insufficient to identify differences among groups, although the PV of the temperature rhythm had a tendency to increase with the severity of the pathology, suggesting a more stable rhythm in peripheral temperature values of depressed patients.

Peripheral temperature can be influenced by activity. However, in our study, we observed that there were differences between day and night for temperature values for the same relative level of activity. This observation suggests that peripheral temperature followed a real endogenous rhythm independent of activity. Healthy subjects demonstrated fewer differences between temperature values during the day and night, which is consistent with lower amplitude values in this variable. This suggested that high activity in healthy people might have masked the temperature rhythm. However, this suggestion is not completely valid, because for all levels of activity, the differences between nocturnal and diurnal values were lowest in the control group (Figure 5).

The peripheral temperature rhythm is partly the result of alternate balances between parasympathetic (vasodilatation) and sympathetic (vasoconstriction) actions in the peripheral skin vessels, which are driven by the SCN [35,36]. Therefore, the lower levels of peripheral temperature during the day in depressed patients could be attributable to higher activity of the sympathetic system. This relationship supports the idea that, in depression, there might be an imbalance in the autonomic system, which could be due to abnormalities in circadian system regulation [37].

Additionally, the results of intrinsic biological rhythms must be interpreted by examining the external rhythms induced by light exposure. The fit of the amplitude of light was highest in the control, indicating that these subjects were exposed to higher levels of light intensity. The amplitude and stability of the light rhythms were lower in the depressive groups than in the control group, which indicated that light may have had less influence on endogenous rhythms in depressed patients than in controls. Our results demonstrated that although the amplitude of light intensity was not different among the groups, the PV of light intensity values was higher in the control group than in the depressive groups, indicating more stability of this variable in the control group. Some studies have demonstrated a relationship between light intensity and seasonal depression [38,39]. One hypothesis that may help explain abnormalities in the regulation of circadian rhythms has suggested that changes in the length of the endogenous period leads to changes in the phase angle of light synchronisation [40]. Therefore, the intensity and period when the subject is exposed are important, as well as the phase and the spectrum.

The simultaneous assessment of light, activity, and temperature rhythms in the current study was essential because all three variables provided information on the functioning of the circadian pacemaker, as well as possible masking due to light exposure, and its effects on the pacemaker. Discriminant analysis provided important information about the capability of chronobiotic parameters to be considered as a tool for the diagnosis and prognosis of depression. The next step in future research is to perform a prospective study to evaluate the sensitivity, specificity, and predictive value of these parameters .

One alternative explanation for our findings related to the changes in chronobiological parameters may be related to the antidepressant treatment used in the chronically depressed patients. One study using an agonist at MT(1)/MT(2) melatonin receptors and an antagonist at 5-HT(2C) serotonin receptors showed a significant difference in the relative amplitude of the circadian rest-activity cycle [41]. Additionally, antidepressant treatments, including tricyclic medications, increase the circadian amplitude of temperature [42]. In our study, the effect of medication could not be clearly considered. Notably, the D_c_ patients were chronic patients who had received medication. However, in spite of their treatment, they scored higher on the depressive scales than did the D_fe_ patients. Therefore, we conclude that, in the current study, alterations in circadian rhythms were due to severity of the pathology and not necessarily to the medication itself.

Limitations due to the design of the study are as follows: (i) this was a cross-sectional study, and therefore, we could not establish a direct cause-consequence relationship; (ii) depressive subjects with more intense symptoms were under treatment; therefore, we could not affirm that the changes in rhythm were due only to the effect of the severity of the symptoms; and (iii) in this study, we assessed the intensity but not the quality of the light.

Finally, this study raises some hypotheses to be tested in longitudinal studies. First, it suggests that in subjects with diseases, there is a shift in circadian rhythm to adapt and maintain the energy needed for survival. Second, the results in this study underscore the theory that the lack of synchronisation between rhythms can be decoded by physiological systems as a stressor. This chronodisruption is capable of starting, accelerating, perpetuating, and exacerbating neuropsychiatric symptoms because the rhythms are integrated into basic cellular endocrine rhythms that build rhythmic, structural, and functional networks.

## Conclusions

In conclusion, we show that the amplitude and stability of circadian rhythms are influenced by depressive states. This result correlates with the severity of the clinical manifestation of depression may be an important tool to be used in clinical set.

## Abbreviations

BDI: Beck Depression Inventory; 5-HT(2C): 5-hydroxytryptamine (serotonin) receptor 2C; HAM-D: Hamilton Depression Scale; HCPA: Hospital de Clínicas de Porto Alegre; MT: Melatonin Receptor; MADRS: Montgomery-Äsberg Scale; PV: Percentage of variance; SRQ-20: Self-Report Questionnaire; SCN: Suprachiasmatic nucleus; Dfe: Depressive-first episode group; Dc: Depressive-chronic group

## Competing interests

The authors declare that they have no competing interests.

## Authors’ contributions

TC, ADN, RL and MPLH designed the study, wrote the protocol, and managed the literature searches and analyses. TC and MPLH wrote the first draft of the manuscript. CM, RS, GD, data collection and literature revision and RL, TC, MPLH make the final revision of the manuscript. All authors contributed to and have approved the final manuscript.

## Pre-publication history

The pre-publication history for this paper can be accessed here:

http://www.biomedcentral.com/1471-244X/13/77/prepub
